# Atypical Guillain-Barre Syndrome With a Sensory Level and Hyper-Acute Presentation: A Case Report

**DOI:** 10.7759/cureus.38262

**Published:** 2023-04-28

**Authors:** Sharath Kommu, Vidyasagar Cirra, Siva Prasad Reddy Pesala, Shalini Arepally

**Affiliations:** 1 Hospital Medicine, Marshfield Clinic Health System, Rice Lake, USA; 2 Neurology, Marshfield Clinic Health System, Marshfield, USA; 3 Family Medicine, Marshfield Clinic Health System, Rice Lake, USA

**Keywords:** hyper-acute gbs, atypical gbs, sensory level, gbs, guillain-barre syndrome

## Abstract

A 46-year-old man with a prior history of cervical spondylosis and myelopathy needing cervical spinal surgery three years back presented to the emergency department with acute onset areflexic flaccid weakness of both lower extremities, with a sensory level at T10. Magnetic resonance imaging studies (MRI) of the cervical, thoracic, and lumbar spine ruled out significant cord compression, spinal cord ischemia, spinal shock, or findings to suggest transverse myelitis. CSF analysis showed normal albumin and protein; however, with the features of paraplegia with flaccidity, areflexia, absence of bowel and bladder symptoms, and MRI ruling out other possibilities, a diagnosis of Guillain-Barre syndrome (GBS) was made. The patient was treated with intravenous immunoglobulin (IVIG) and showed a clinical response, with improvement in strength in both lower extremities. This case is rare and unique, as it exhibits atypical features for a GBS case, including a sensory level and hyper-acute presentation, with the onset of weakness to a nadir within an hour. This case highlights the importance of awareness of such atypical GBS presentations so that the diagnosis is not missed and is appropriately managed for favorable patient outcomes.

## Introduction

Acute immune-mediated polyneuropathies or Guillain-Barre syndrome (GBS) is one of the causes of acute-onset flaccid weakness. It is caused by an immune-mediated neuronal injury resulting in demyelination (acute inflammatory demyelinating polyneuropathy or AIDP) [1-3] or axonal loss (acute motor axonal neuropathy or AMAN and acute motor and sensory axonal neuropathy or AMSAN) [1]. This immune response can be triggered by infections: gastrointestinal infections like Campylobacter jejuni gastroenteritis [4]., respiratory tract infections like influenza, coronavirus disease 2019 (COVID-19) [5], etc., vaccinations like influenza vaccination, recombinant zoster vaccination, and other triggers like surgery, trauma, lymphoma, medications like isotretinoin, immune checkpoint inhibitors, etc. The symptoms usually manifest in a few days to a week and reach nadir by around four weeks of onset. Patients typically have ascending flaccid paralysis with hyporeflexia. Sensory involvement is common, and some patients can have dysautonomia. GBS presenting with a sensory level is rare, and the presence of a sensory level usually suggests an alternative diagnosis. While this syndromic manifestation will help diagnose GBS, cerebrospinal fluid (CSF) analysis, nerve conduction studies (NCS), and electromyogram (EMG) can aid in supporting the diagnosis.

## Case presentation

A 46-year-old man has a history of cervical spondylosis and myelopathy, managed with a posterior spinal fusion of C3 to T1 with decompressive laminectomies of C3 to C7 in November 2020. Despite the surgery, he had residual 4/5 strength (per the medical research council (MRC) muscle strength scale) in his right upper and lower extremities. He presented to the emergency department in March 2023 with an acute onset of weakness and numbness in both lower extremities with muscle strength of 0/5 by the MRC scale, which occurred rapidly over one hour. He retained his bowel and bladder function. He denied any recent infection or vaccinations.

**Figure 1:**
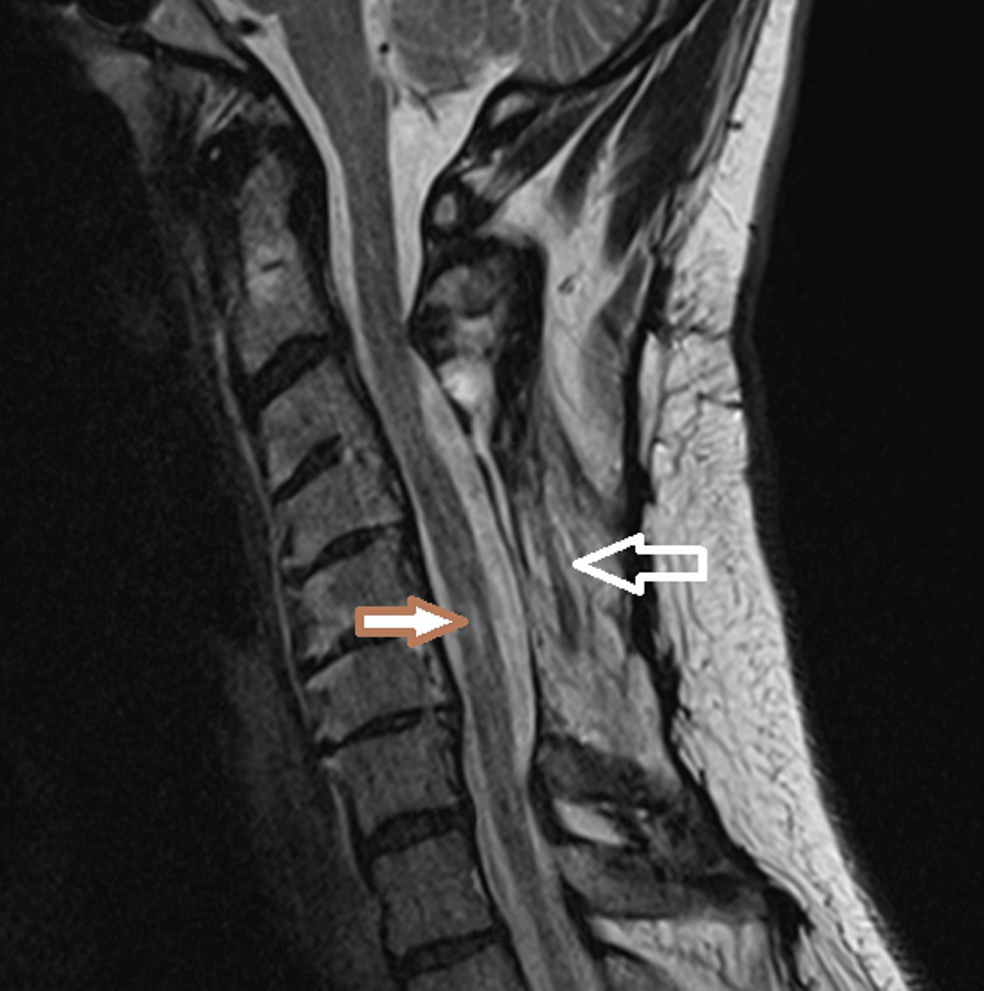
MRI cervical spine, sagittal view T2-weighted image, showing chronic postoperative changes related to previous procedures of posterior spinal fusion and decompressive laminectomies (white arrow) and area of myelomalacia at C5-C6 level (brown arrow) MRI - magnetic resonance imaging

**Figure 2:**
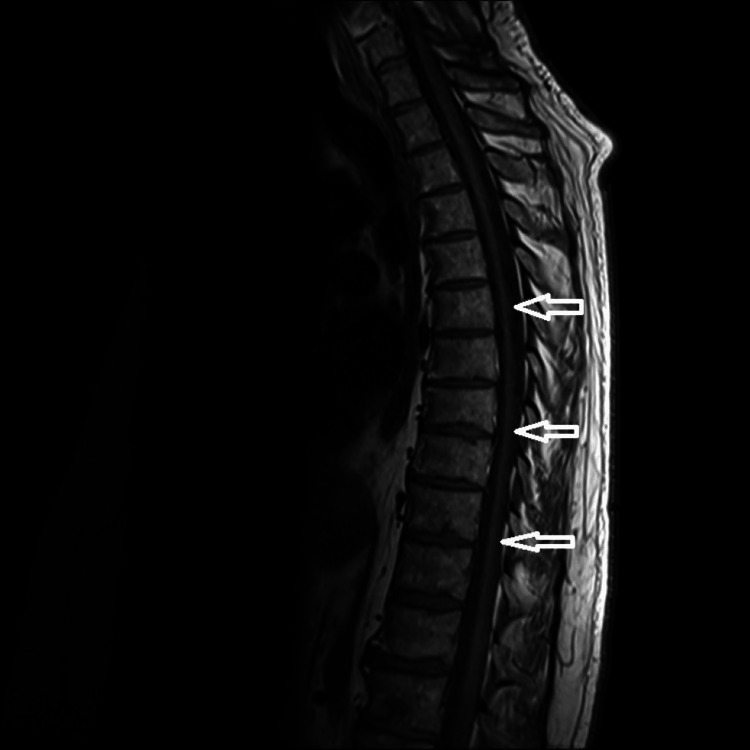
MRI thoracic spine, sagittal view, T1-weighted image, showing normal spinal cord (white arrows) without cord compression or signal changes. MRI - magnetic resonance imaging

**Figure 3:**
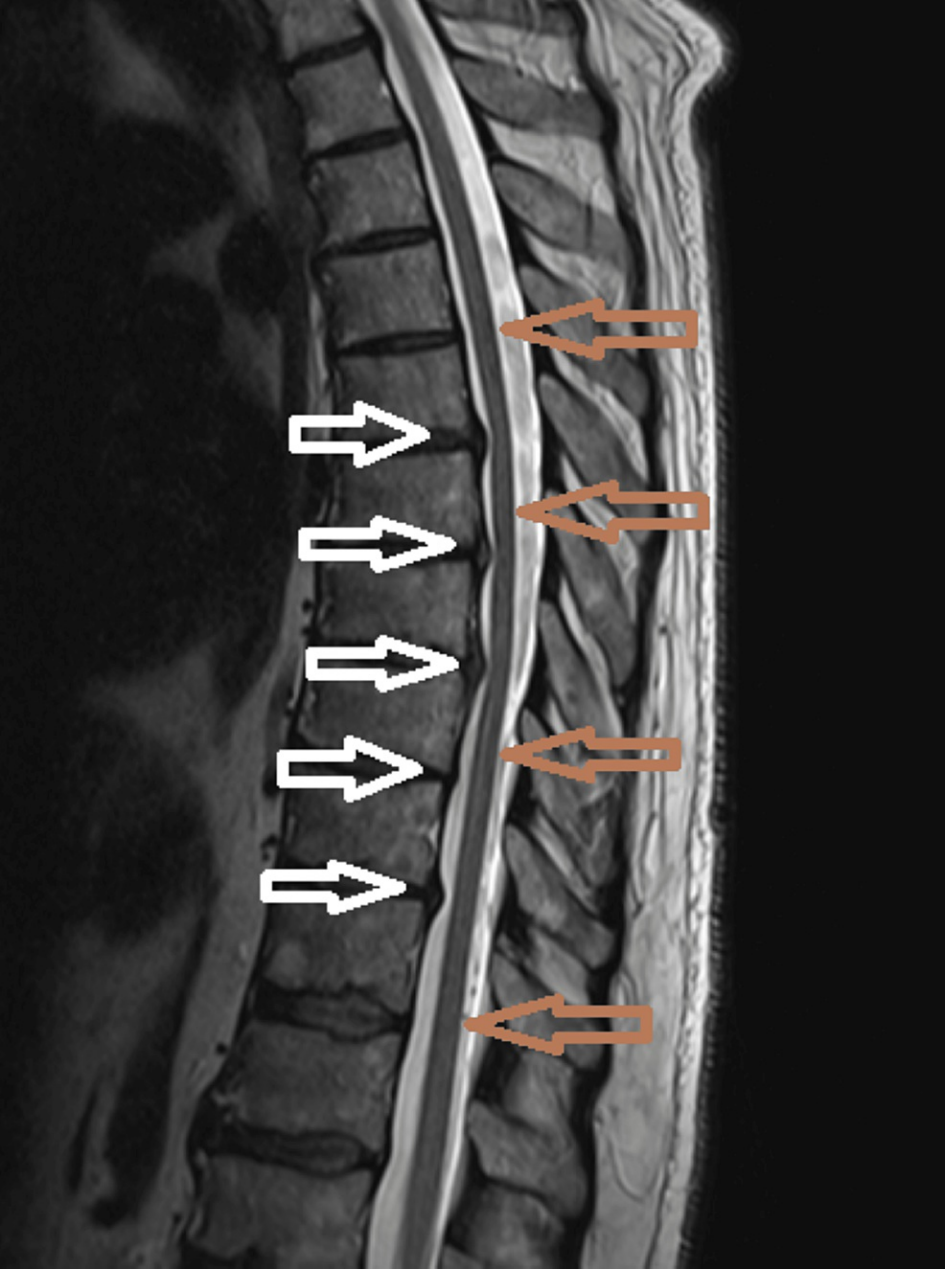
MRI thoracic spine, sagittal view, T2-weighted image, showing disc protrusions (white arrows), without cord compression and normal cord (brown arrows), with no signal changes. MRI - magnetic resonance imaging

A physical examination revealed that the patient had complete motor paralysis of both the lower extremities with flaccidity, absent bilateral knee and ankle reflex, along with a sensory level of T10 with absent sensation to touch, pain, vibration, and proprioception. CSF analysis showed normal albumin and protein; the values of the CSF analysis are shown in Table 1. Magnetic resonance imaging (MRI) of the cervical spine showed chronic changes of myelomalacia at C5 and C6 with expected chronic postoperative changes related to posterior spinal fusion and decompressive laminectomies (Figure 1). MRI of the thoracic spine (Figure 2 and Figure 3) showed T6 to T11 disc protrusions with no cord compression and no signal changes in the cord on T2-weighted images. MRI of the lumbar spine also showed evidence of disc protrusions with no significant central canal stenosis.

**Table 1:** Findings of cerebrospinal fluid analysis CSF - cerebrospinal fluid. WBC - white blood cell, RBC - red blood cell, IGG - immunoglobulin G

CSF parameter	Result	Normal range
Color	Colorless	Colorless
RBC	0/µL	0/µL
WBC	2/µL	0-5/µL
Protein	42 mg/dL	15-45 mg/dL
CSF Synthesis Rate	<1.0 mg/24hr	0.0-8.0 mg/24hr
CSF IGG Index	0.53	0.30-0.70
CSF IGG/Albumin	0.09	0.00-0.27
CSF-Albumin	23.7 mg/dL	15.0 – 32.0 mg/dL
CSF IGG	2.2 mg/dL	0.0 – 6.6 mg/dL
CSF Glucose	71 mg/dL	40-70 mg/dL

A neurologist was consulted, and based on the findings of areflexic flaccid paralysis, sparing of bowel and bladder function, CSF analysis, and MRI findings ruling out other possibilities, a diagnosis of GBS was suspected, and the patient was started on intravenous immunoglobulin (IVIG) at a dose of 400 mg/kg per day. During the five days of treatment with IVIG, the patient showed progressive improvement. By the fifth day, the strength in both lower extremities gradually improved to 4/5, though not back to his baseline. He could ambulate with standby assistance and was discharged home with advice for outpatient physical therapy to help him regain his strength and mobility.

## Discussion

The differential diagnoses, in this case, included transverse myelitis, acute cord ischemia, spinal shock, cord compression, and atypical presentation of GBS. With flaccid weakness in both lower extremities with no progression to spasticity, low CSF immunoglobulin G (IGG) index and absence of pleocytosis in CSF analysis, and the absence of T2 hyperintensity in the MRI, the possibility of transverse myelitis was ruled out. With the rapid onset of weakness, one might suspect spinal ischemia; however, an MRI of the thoracic spine, as reviewed by the neuro-radiologist, showed no signal changes in the cord to suggest cord ischemia. Spinal shock is another differential diagnosis because of the rapid onset of symptoms. However, with the absence of potential triggers for spinal shock (like trauma, hypotension, bradycardia, etc.), sparing of bowel and bladder function, and the lack of signal changes in the thoracic spinal cord in the MRI, the possibility of spinal shock was ruled out. No cord compression is seen in MRI images of the thoracic spine, ruling out that possibility as well.

With the patient having areflexic flaccid weakness in the lower extremities, sparing of bowel and bladder function, and other possibilities ruled out, the case of GBS was high in the differential diagnosis. GBS is characterized by elevated CSF protein and albumin; however, they may not be elevated when tested earlier in the course of illness, which appears to be the finding in this case. The patient was treated with a five-day course of IVIG and showed gradual improvement. The treating hospital, a small community hospital, did not have the diagnostic modality of a nerve conduction study, which could further aid the diagnosis and prognosis. However, the findings on the physical exam, collaborative evidence from CSF analysis and MRI of the spine, and the response to therapy helped us exclude other possibilities and conclude the diagnosis of GBS.

National Institute of Neurological Disorders and Stroke (NINDS) diagnostic criteria are typically used in clinical practice to diagnose GBS. However, we have to understand that there could be outliers. A study by Govoni et al. [6] found that 84% of patients diagnosed with GBS fulfilled the NINDS criteria, and 16% did not. The case discussed here is interesting and rare, as GBS typically does not present with a sensory level, and the patient discussed here had a sensory level at T10. On reviewing the literature, GBS with a sensory level is extremely rare, and only a few cases have been reported. Alfahad and Kelly (2014) reported a pure T7 sensory level case as an isolated manifestation of GBS [7]. Khoo et al. (2018) reported a case of GBS presenting with acute urinary retention and T6 sensory level [8]. Al-Ameen et al. (2022) reported a case of atypical GBS with a T6 sensory level, where the patient had sudden onset numbness and tingling in the lower abdomen and lower limbs [9]. Agarwal and Fernandez Bowman (2020) reported a case of synchronous occurrence of GBS and transverse myelitis of unknown etiology in an adolescent, where the patient presented with ascending motor weakness and T6 sensory level [10].

In addition to the presence of a sensory level, unlike all the other case reports with a sensory level, where the symptoms develop gradually over a few days, in this case, the symptoms progressed rapidly with the onset of weakness to a nadir within one hour. Hyper-acute GBS refers to the cases in which the nadir is reached in one to two days [11]. Hyper-acute GBS is rare [11] and only recorded in a few case reports and case series [12-15], where the time to reach the nadir ranged from 12 to 48 hours. In our case, the nadir was reached within one hour, which is unique. We suspect that the patient's history of cervical myelopathy with chronic underlying weakness may have contributed to the rapid progress of the weakness.

To our knowledge, this is the first case of GBS to be reported, which has a sensory level and hyper-acute presentation, with the onset of weakness to a nadir within one hour. In addition, our patient had previous cervical spondylosis and myelopathy, complicating the presentation, which makes this case even more unusual. He was treated appropriately with IVIG and showed signs of recovery. This case highlights the importance of early identification of such atypical manifestations of GBS, as treatment started promptly can result in good patient outcomes.

## Conclusions

GBS can present with atypical features. Good history taking, thorough neurological examination, and collaborative evidence with CSF analysis, MRI, and nerve conduction studies can aid in identifying or ruling out other potential causes. This case emphasizes the need for awareness among clinicians about the possibility of such rare manifestations of GBS, as a timely treatment institution can result in favorable results. Further studies are needed to explore and identify the incidence of such rare manifestations of GBS and their underlying mechanisms.
